# Systemic Therapy Is Associated with Improved Oncologic Outcomes in Resectable Stage II/III Intrahepatic Cholangiocarcinoma: An Examination of the National Cancer Database over the Past Decade

**DOI:** 10.3390/cancers14174320

**Published:** 2022-09-03

**Authors:** Rebecca Marcus, Wade Christopher, Jennifer Keller, Sean Nassoiy, Shu-Ching Chang, Melanie Goldfarb, Ronald Wolf, Zeljka Jutric

**Affiliations:** 1 Department of Surgical Oncology, Providence Saint John’s Cancer Institute, Santa Monica, CA 90404, USA; 2 Medical Data Research Center, Providence Saint Joseph Health, Portland, OR 97229, USA; 3 Division of Hepatobiliary and Pancreas Surgery, University of California Irvine, Orange, CA 92868, USA

**Keywords:** intrahepatic cholangiocarcinoma, neoadjuvant therapy, adjuvant therapy, National Cancer Database

## Abstract

**Simple Summary:**

Intrahepatic cholangiocarcinoma (ICC) is a primary liver cancer that currently has limited treatment options and an overall poor prognosis. Evidence-based guidelines for the management of resectable ICC are lacking. We investigated three treatment strategies for resectable ICC using a large cancer registry and compared their use and oncologic outcomes. Our findings suggest a benefit of both neoadjuvant and adjuvant therapy for patients with high-risk resectable ICC. Prospective and randomized studies are needed to better define patients who may benefit from systemic therapy and to clarify the most appropriate sequencing of treatment for resectable ICC.

**Abstract:**

Limited evidence-based management guidelines for resectable intrahepatic cholangiocarcinoma (ICC) currently exist. Using a large population-based cancer registry; the utilization rates and outcomes for patients with clinical stages I-III ICC treated with neoadjuvant chemotherapy (NAT) in relation to other treatment strategies were investigated, as were the predictors of treatment regimen utilization. Oncologic outcomes were compared between treatment strategies. Amongst 2736 patients, chemotherapy utilization was low; however, NAT use increased from 4.3% to 7.2% (*p* = 0.011) over the study period. A higher clinical stage was predictive of the use of NAT, while higher pathologic stage and margin-positive resections were predictive of the use of adjuvant therapy (AT). For patients with more advanced disease, the receipt of NAT or AT was associated with significantly improved survival compared to surgery alone (cStage II, *p* = 0.040; cStage III, *p* = 0.003). Furthermore, patients receiving NAT were more likely to undergo margin-negative resections compared to those treated with AT (72.5% vs. 62.6%, *p* = 0.027), despite having higher-risk tumors. This analysis of treatment strategies for resectable ICC suggests a benefit for systemic therapy. Prospective and randomized studies evaluating the sequencing of treatments for patients with high-risk resectable ICC are needed.

## 1. Introduction

Intrahepatic cholangiocarcinoma (ICC) is an aggressive primary hepatic malignancy with increasing incidence in many parts of the world and overall poor prognosis [1,2,3]. Currently, 5-year overall survival (OS) rates for ICC remain <5% [4,5]. Surgical resection represents the only potential curative treatment for ICC; however, the majority of patients present with advanced disease at diagnosis [6,7]. Up to one-third of patients initially thought to be eligible for curative-intent resection are found to have non-resectable diseases at the time of surgery [8,9,10]; moreover, overall, only 15% of patients are eligible for curative resection [8,11]. Treatment failure is common amongst patients who undergo curative-intent resection, with recurrences in as many as 66% of patients and median OS of only 28–36 months [4,11,12].

While systemic therapy has been investigated for improvements in patient outcomes, there remains a paucity of level one evidence to guide the management of ICC and a lack of consensus regarding the appropriate treatment regimen for patients with resectable disease. The current National Comprehensive Cancer Network (NCCN) guidelines recommend adjuvant therapy (AT) following curative-intent surgeries in ICC patients as the preferred treatment regimen [13]. This is particularly important for patients at high risks of recurrence, including those with large tumors, regional nodal involvement, and microscopically positive margins [12,14,15,16]. These recommendations are based on a limited number of randomized clinical trials yielding conflicting results, most prominently the BILCAP study [17]. Unsurprisingly, AT has yielded only a modest survival benefit to-date [18,19].

Within the past decade, there has been a trend toward the increased use of neoadjuvant therapy for many GI malignancies [20,21,22,23]. The application of NAT to an oncologic treatment regimen provides potential advantages of tumor downsizing, conversion of unresectable to resectable disease, and increased R0 resection rates [24,25,26]. NAT also potentially allows for a more appropriate selection of patients who may or may not benefit from surgical intervention [11]. Lastly, the performance of NAT as opposed to AT helps ensure the receipt of multimodality treatment, which is crucial given that a significant proportion of patients undergoing complex hepatobiliary surgery ultimately do not receive and/or complete their intended AT [27,28].

As the use of NAT for ICC has not been well-studied, we sought to evaluate the role of NAT for resectable ICC as part of a multidisciplinary oncologic treatment regimen. We used the National Cancer Database (NCDB) with the aim to characterize utilization rates of ICC treated with NAT in relation to other treatment strategies (i.e., surgery alone and AT), to describe the clinicopathologic characteristics associated with NAT use, and to examine the effect of NAT on OS in patients with resectable ICC.

## 2. Materials and Methods

### 2.1. Study Design

Using data from the NCDB, a cohort of patients diagnosed with intrahepatic cholangiocarcinoma (ICC) between 2006 and 2016 was identified. The NCDB is a large, hospital-based cancer registry established in 1989 and jointly maintained by the Commission on Cancer (CoC) of the American College of Surgeons and the American Cancer Society. Annually, more than 1500 CoC hospitals contribute to the database, which currently represents more than 70% of newly diagnosed cancers [29]. Reporting facilities are required to have at least 90% patient follow-up. This study protocol was reviewed and deemed exempt by the guidelines set forth by the Institutional Review Board at Saint John’s Cancer Institute. All data were deidentified and the NCDB was not asked to verify the results or the statistical validity of this study.

### 2.2. Study Cohort

**Figure 1:**
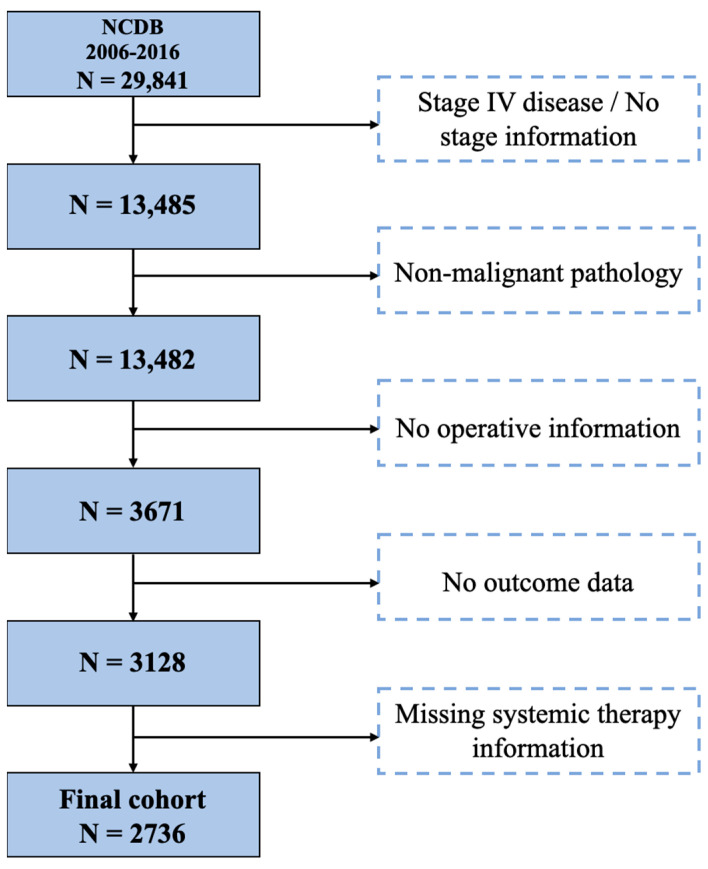
CONSORT flow chart for the selection criteria of patients with intrahepatic cholangiocarcinoma included in the study population derived from the National Cancer Database. CONSORT: consolidated standards of reporting trials.

The study population was restricted to patients greater than 18 years with ICC. Using the 3rd edition of the World Health Organizations’ International Classification of Disease, ICC was defined by topographic code C22.1 and morphological codes 8140 and 8160. Our analyses were restricted to patients with stages I–III disease, as these patients were considered potential candidates for curative-intent resection. Exclusion criteria included non-malignant tumors, stage IV disease, patients with no data on their received operative procedure (e.g., wedge resection, segmentectomy, hemi-hepatectomy, and extended hepatectomy), those for which information regarding the sequence of systemic therapy administration and surgery performance was missing, and those with no outcome data (Figure 1).

### 2.3. Variables and Outcomes

The receipt of neoadjuvant therapy was the principal variable of interest and was defined as chemotherapy alone or combined chemotherapy and radiation therapy prior to surgical resection. Patients were stratified by treatment strategy into three cohorts: surgery alone, neoadjuvant therapy followed by surgery (NAT), or surgery followed by adjuvant therapy (AT). Patient and tumor-specific information were extracted from the NCDB. The primary outcome of interest was overall survival (OS), which was measured as the time from diagnosis until death or last follow-up.

### 2.4. Statistical Analysis

Descriptive statistics were used to summarize the distributions of patient- and tumor-specific variables. Baseline characteristics were compared using Chi-squared tests and ANOVA analyses for categorical and continuous variables, respectively. Temporal trends in the use of individual treatment strategies were plotted and compared. Multivariable logistic regression analyses including age, gender, race, year of diagnosis, Charlson comorbidity index (CCI), insurance status, facility type/location, city type, tumor size, clinical stage, and the receipt of radiation as part of the treatment regimen were performed to examine factors that contributed to patients receiving NAT. Similar analyses were performed to determine factors predictive of receiving AT, including all previously listed variables with the exception of clinical stage, and additional pathological variables including tumor grade, pathologic stage, type of surgery, performance of lymphadenectomy, and surgical margin. We also assessed the association of treatment strategy with negative surgical resection margins (R0 vs. R1/2) using multivariable logistic regression analyses adjusted for age, gender, race, year of diagnosis, CCI, insurance status, facility type/location, city type, receipt of radiation as part of the treatment regimen, tumor size, tumor grade, clinical stage, type of surgery, and the performance of lymphadenectomy. Of note, two editions of the American Joint Committee on Cancer (AJCC) staging system for ICC were in use during the study time span: the 6th edition from 2006 to 2009 and 7th from 2010 to 2016. To minimize misclassification bias from differing staging criteria, all staging information was re-coded to reflect the current AJCC 8th edition using a previously described framework [30].

The OS for patients undergoing NAT, surgery alone, and AT was compared using the Kaplan–Meier method with log-rank tests. To examine the independent effect of treatment strategy and the interactive effect of clinical stages by treatment strategies on OS, multivariable Cox proportional-hazards regressions were performed and adjusted for the year of diagnosis, patient demographics, and tumor characteristics. Cox proportional-hazards regression with an inverse probability of treatment weighting (IPTW) analyses by clinical stages was also performed and compared. The stabilized inverse probability weights were derived from the predicted probabilities of treatment strategy on the same set of covariates, based on the average treatment effects among the patients receiving NAT and generalized boost models using R package “twang.” [31]. To minimize potential immortal time bias, we further conducted three-month landmark analyses where only patients who survived at least three months after ICC diagnosis were included [32]. The rationale for our choice of a three-month landmark was that, on average, NAT patients take three months longer to undergo surgery compared to patients undergoing upfront surgical resection. A *p* value < 0.05 was considered statistically significant. All statistical analyses were conducted with R, version 3.6.3.

## 3. Results

### 3.1. Patient Demographics and Clinical Profile

2736 patients met the inclusion criteria and were included in our analyses. Table 1 summarizes patient sociodemographic, clinical, tumor-related, and treatment-related factors by treatment strategy received. For the complete study cohort, the majority of patients were white, non-Hispanic (78.4%), females (55.6%), and with private insurance (39.5%) or Medicare (56.3%). The mean age at diagnosis was 64.5 years (SD: 10.9) and median follow up was 30.8 months (IQR: 16.6–53.0) (Supplementary Table S1).

### 3.2. Use of Chemotherapy

**Figure 2:**
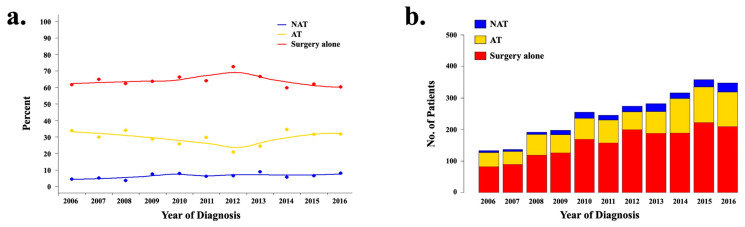
Among patients with resectable intrahepatic cholangiocarcinoma, (**a**) treatment strategy utilization patterns and (**b**) the annual proportion of patients receiving neoadjuvant chemotherapy (NAT), adjuvant chemotherapy (AT), and surgery alone over time.

Overall, chemotherapy utilization was low, with 63.9% of patients undergoing surgery alone (Figure 2a). 182 (6.7%) patients received NAT, and 805 patients (29.4%) received AT. Among patients who received chemotherapy (NAT or AT; *n* = 987), the majority received a multidrug regimen (*n* = 479, 48.5%), and the use of multidrug regimens increased over the study period from 13.4% to 21.2% (*p* < 0.001). Patients receiving AT had higher rates of radiation use when compared to those patients who received either surgery alone (38.5% vs. 3.4%; *p* < 0.001) or NAT (38.5% vs. 20.9%; *p* < 0.001). Overall, the rate of radiation utilization decreased during the study period from 20.8% to 13.6% (*p* < 0.001).

The mean age of patients who received NAT was 60.4 years (SD: 10.9) versus 66.1 years (SD: 10.7) for those treated with surgery alone (*p* < 0.001) and 61.9 years (SD: 10.9) for those treated with AT (*p* = 0.104). In general, patients who received NAT tended to have been diagnosed later in the study period (*p* = 0.011) and received their treatment at academic/research facilities (*p* < 0.001). Compared to both the surgery only and AT cohorts, those patients who underwent NAT were younger (*p* < 0.001) and healthier (CCI 0–1; *p* < 0.001) with larger tumors (size > 5 cm; *p* < 0.001) and more advanced clinical T stage (*p* < 0.001). Patients receiving NAT did not have significantly more advanced clinical N stage (*p* = 0.186) compared to patients receiving other treatment strategies (Table 1).

Comparing NAT and AT treatment strategies only, patients who received NAT were more likely to have larger tumors (*p* < 0.001) and higher clinical T stage (*p* = 0.028), yet they were also more likely to undergo margin-negative resections (72.5% vs. 62.6%, *p* = 0.027). We also observed a trend toward higher clinical stages in patients receiving NAT compared to AT (*p* = 0.071). Finally, the rate of lymphadenectomy performance was relatively low throughout the study period regardless of treatment strategies. Specifically, almost half (49.3%) of patients did not undergo lymph node resection. For those who did, only 10.8% of patients had six or more lymph nodes harvested (Table 1).

### 3.3. Trends over Time and Predictors of NAT Utilization

182 (6.7%) patients received NAT. Throughout the study period, the utilization of NAT increased significantly from 4.3% to 7.2% (*p* = 0.011), and the rates of multidrug regimen utilization increased from 16.7% to 100% (Figure 2b). Multivariable logistic regression analyses identified the year of diagnosis (2013–2016 vs. 2006–2008: OR 2.41, 95% CI 1.37–4.24; 2009–2012 vs. 2006–2008: OR 2.09, 95% CI 1.19–3.68), female sex (OR 1.46, 95% CI 1.05–2.04), treatment at an academic/research facility (OR 1.84, 95% CI 1.26–2.69), larger tumor size (>5 cm vs. <2 cm: OR 3.80, 95% CI 1.34–10.72), and increased clinical stage (cStage II vs. I: OR 1.84, 95% CI 1.22–2.78; cStage IIIB vs. I: OR 3.28, 95% CI 1.30–8.24) as predictors of NAT utilization (Table 2). Conversely, patients with increased age were less likely to receive NAT (<55 vs. 55–69 years: OR 0.55, 95% CI 0.36–0.83; <55 vs. 70+ years: OR 0.20, 95% CI 0.12–0.34), and there was a trend toward a lower likelihood of NAT in patients with CCI >1 (*p* = 0.076) (Table 2). We did not find a statistically significant association between the receipt of radiation and NAT utilization.

### 3.4. Trends over Time and Predictors of AT Utilization

805 (29.4%) patients received AT during the study period, and the utilization of AT did not change significantly during this time (Figure 2a,b); however, the use of multidrug AT regimens did increase over time from 24.4% to 51.8% (*p* = 0.003). Comparing the treatment strategy of AT to surgery alone, multivariable logistic regression analyses identified female sex (OR 1.43, 95% CI 1.16–1.75), larger tumor size (>5 cm vs. <2cm: OR 1.93, 95% CI 1.21–3.07), positive surgical resection margins (OR 1.83, 95% CI 1.41–2.39), and increased pathologic stage (pStage II vs. I: OR 2.50, 95% CI 1.87–3.34; pStage IIIB vs. I: OR 3.33, 95% CI 1.94–5.72) as predictors of AT utilization (Table 3). Conversely, patients with increased age (<55 vs. 55–69 years: OR 0.69, 95% CI 0.51–0.92; <55 vs. 70+ years: OR 0.39, 95% CI 0.28–0.55) and treatment at an academic/research facility (OR 0.69, 95% CI 0.55–0.86) were less likely to receive AT. There was also a trend toward a lower likelihood of AT in patients with CCI >1 (*p* = 0.107) (Table 3). We did not find a statistically significant association between tumor grades or performances of lymphadenectomy and AT utilization.

### 3.5. Overall Survival Analyses

**Figure 3:**
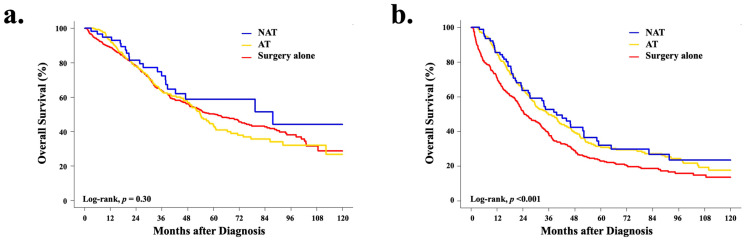
Unadjusted Kaplan–Meier curves for overall survival by treatment strategy for patients with (**a**) clinical stage I (*n* = 1072) and (**b**) clinical stages II-III (*n* = 760) resectable intrahepatic cholangiocarcinoma.

The median follow-up for the study cohort was 30.8 months (IQR: 16.6–53.0). The median and 5-year overall survival (OS) for the cohort were 41.6 months (95% CI 39.6–45.4) and 39.4% (95% CI 37.3–41.5), respectively. The OS was similar across treatment strategies, with OS for the NAT cohort being slightly longer (42.3 months, 95% CI 34.4–58.1), followed by surgery alone (OS 41.7 months, 95% CI 39.3–46.3) and AT (41.1 months, 95% CI 37.2–46.9); however, there was no statistically significant difference between treatment strategies (log-rank, *p* = 0.800) (Figure 3a). Interestingly, when OS analyses were stratified by clinical stage, for both stage II and III disease, the receipt of systemic therapy (either NAT or AT) was associated with significantly improved survival compared to surgery alone (cStage II, *p* = 0.040; cStage III, *p* = 0.003) (Figure 3b).

**Figure 4:**
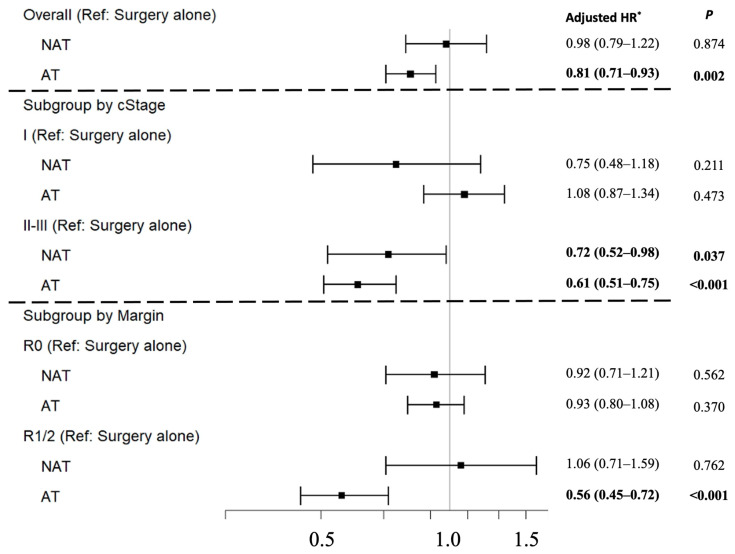
Comparison of overall survival by treatment strategy using risk-adjusted multivariable Cox proportional-hazards regression analyses for patients with resectable intrahepatic cholangiocarcinoma. * Model adjusted for year of diagnosis, age, gender, race, insurance status, Charlson comorbidity index, average income, average level of education, facility type and location, population density, tumor size, tumor grade, pathologic stage, surgical type, performance of lymphadenectomy, and inclusion of radiation in the treatment regimen.

In risk-adjusted multivariable Cox proportional-hazards regression analyses, for clinical stage II and III diseases, the association of NAT or AT with improved an OS remained significant when compared to surgery alone (NAT: adjusted hazards ratio (aHR) 0.72, 95% CI 0.52–0.98; AT: aHR 0.61, 95% CI 0.51–0.75) (Figure 4). However, for clinical stage I diseases, the treatment strategy had no significant association with OS (Figure 4). Other factors found to be associated with an increased risk of death on multivariable analyses included male gender, increased age and CCI, larger tumor size, higher tumor grade, and margin-positive resections (Supplementary Figure S1). The overall risk of death from ICC decreased over the study period. Findings based on three-month conditional landmark analyses in both multivariable Cox proportional-hazards regression analyses and Cox regression with IPTW using propensity score method remained similar (Supplementary Figure S2).

**Figure 5:**
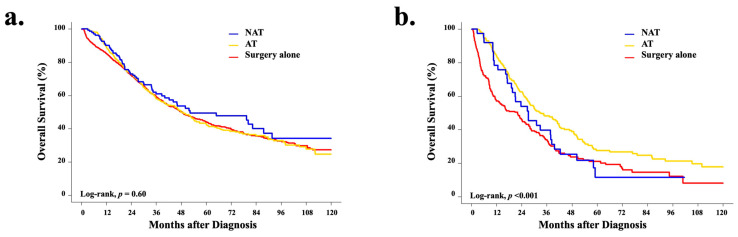
Unadjusted Kaplan–Meier curves for overall survival by treatment strategy for patients who underwent (**a**) R0 resection (*n* = 2082) and (**b**) R1 or R2 (*n* = 491) resections for intrahepatic cholangiocarcinoma.

Finally, subgroup analyses based on surgical resection margin (R0 vs. R1/2) were performed. For patients who underwent R0 resections, neither the receipt of NAT nor AT was associated with significantly improved survival compared to surgery alone (Figure 5a). For patients who underwent R1 or R2 resections, only the receipt of AT was associated with significantly improved OS compared to surgery alone (Figure 5b). Furthermore, after adjusting for other risk factors of death, AT remained significantly associated with improved OS compared to surgery alone for patients undergoing R1/2 resections (aHR 0.56, 95% CI 0.45–0.72); the association remained non-significant for patients who had R0 resections (aHR 0.93, 95% CI 0.80–1.08). Subgroup analyses based upon tumor size (≤5 vs. >5 cm) and the performance of lymphadenectomy did not reveal survival differences based on the received treatment strategy.

## 4. Discussion

Surgical resection is currently the first-line treatment for resectable ICC [13]. Systemic therapy, radiation, and various liver-directed therapies are considered in select cases for patients who are high-risk surgical candidates or initially unresectable [25,33,34,35]. Available data on the use of NAT for ICC are limited and often extrapolated from studies utilizing NAT to downstage patients for surgical resection. Other data come from case reports, single institution studies, systematic reviews, or meta-analyses on ICC or from studies examining the utilization of NAT for other GI malignancies [24,36,37]. In this study, we queried the NCDB to examine the current use and associated outcomes of NAT for the treatment of resectable ICC.

We found that the majority of patients with ICC are treated with surgery alone (63.9%), consistent with other published data [11,20,38]. In our study, approximately 30% of patients received AT, and this did not change over time despite the current NCCN guidelines’ preferred recommendation for the receipt of AT in resected ICC patients [13]. The reason for omitting AT is not available within the NCDB, but the most common historically reported explanations include physician recommendations against AT based on patient risk factors and/or patient inability to receive AT due to poor performance status following major hepatic resection. Despite the generally low utilization rates, we found that amongst those patients who did receive AT, the use of multidrug regimens increased significantly over time, a finding that mirrors treatment regimen trends observed for other GI malignancies [39,40,41].

With respect to overall survival (OS), we found a benefit for patients receiving AT compared to surgery alone amongst those with stage II or III disease, but not stage I. We also found a survival benefit with receipt of AT for patients who underwent R1/R2 resection, but not for those who underwent R0 resections. This study uses the most recent AJCC staging system (8th edition), which describes stage I as tumors ≤5 cm (stage Ia) or >5 cm (stage Ib) without vascular invasion [42]. Patients who were stage I and underwent resection with negative margins did not experience longer OS when treated with AT compared to surgery alone. Our findings are consistent with previously published studies [18,43], but given the mixed results reported in the literature and the retrospective nature of the current study, future prospective trials are needed to investigate this finding further.

The total number of patients receiving NAT was very low (6.7%). This is consistent with other published series, including case reports, retrospective analyses, systematic reviews, and meta-analyses, many of which were aimed at downstaging patients who were not initially resectable [24,36,37]. Data from our study support the idea that the use of NAT is limited to patients considered to be at high risks of recurrence. We found that patients who received NAT were diagnosed later in the study period and were more likely to have received their treatment at academic/research facilities. They were also younger and had larger tumors with more advanced clinical stages. The utilization rate of NAT multidrug regimens was found to increase over time in our study, which is consistent with trends seen in treatment regimens utilized for other GI malignancies [21,41,44].

Another important finding from our study is that patients receiving NAT had higher R0 resection rates compared to the AT cohort, despite these patients having larger and more advanced clinical stage tumors. Given that in current clinical practice the majority of patients treated with NAT are receiving it because they are considered high risk, it is intriguing that these patients still achieved high rates of R0 resections and longer OS. In fact, patients receiving NAT had the longest OS in our study, albeit this difference was not statistically significant. Considering the low utilization of NAT nationally and its potential benefits, these findings should serve as the foundation for future prospective and randomized trials designed to generate necessary data to establish guidelines for the sequencing of treatment for resectable ICC. The results of the current study also suggest a potential benefit of NAT in resectable higher clinical stage ICC, which is similar to findings demonstrated for other GI malignancies [41,45,46].

Similarly to AT, an OS benefit of NAT was found in patients with clinical stages II and III disease. There was no OS benefit for clinical stage I patients. Based on the findings from this study and other published data, it may be the case that treatments with either NAT or AT provide a benefit to high-risk patients, whereas surgery alone can be considered for clinical stage I patients. Prospective studies are currently underway to answer this question. The NCT03579771 trial examining the use of NAT for resected ICC has now been completed and publication of the results are forthcoming [47]. These are anticipated to contribute significantly to the existing ICC literature and to achieving consensus guidelines for the treatment of resectable ICC.

This study has several limitations, including many that are inherent to the retrospective nature of the NCDB. These include coding errors, missing data, and the failure to incorporate several variables that are important when studying ICC (e.g., molecular markers, presence of multifocal tumors, and other high-risk features). The NCBD does not provide sufficiently granular patient and tumor-specific data that may be considered in decision making regarding treatments with NAT or AT. Furthermore, the NCBD does not include information on individual provider practice patterns, patient’s access to multidisciplinary care teams, and hospital resource availability, all of which may impact the decision to utilize systemic therapy. Finally, because treatment decisions are not randomized, subgroup survival differences observed in the NCDB may not be related to treatment strategies, even in the presence of risk adjustments. While propensity score matching can be utilized to overcome some of these limitations, in this particular study, propensity matching was not possible due to the size of the NAT cohort. Nevertheless, the NCDB remains one of the most robust registries with relevant information to examine treatment patterns and outcomes for multiple oncologic patient populations.

Other limitations of this study include the use of OS as the primary outcome of interest, as opposed to disease free survival (DFS), and the inability to perform intention-to-treat analyses (ITT). DFS cannot be calculated within the constraints of the available data within the NCDB. However, because both DFS and OS for ICC patients tend to be quite short, OS was felt to be a reasonable measure of patient outcomes. Moreover, within the constraints of available information in the NCDB, we were unable to perform ITT analyses to account for patients who received NAT but did not go on to receive surgical resection. We performed three-month landmark analyses and Cox regressions with inverse probability of treatment weighting (IPTW) to control for selection and immortal time biases (Supplementary Figure S2). The fact that our OS findings remained significant following these analyses supports that these findings may be related to differences in treatment strategies as opposed to potential confounding factors that are not controlled for. Randomized data are needed to confirm these findings.

## 5. Conclusions

The utilization of NAT for ICC in the United States is low (6.7%), and more commonly seen amongst patients with larger, more clinically advanced tumors. NAT demonstrates potential value in achieving margin-negative resections. Furthermore, both NAT and AT demonstrate OS benefits for patients with stage II and III disease. Patients with stage I ICC do not demonstrate a benefit from either NAT or AT. Prospective and randomized studies are needed for an improved definition of patient subgroups that may benefit from systemic therapy and to further clarify the most appropriate sequencing of treatment for resectable ICC.

## Figures and Tables

**Table 1:** Baseline demographic and tumor characteristics by treatment strategy. Abbreviations as follows, NAT: neoadjuvant therapy; AT: adjuvant therapy.

	Treatment Strategy			
Characteristic, *N* (%)	Surgery Alone	NAT	AT	*p* Value
Year of diagnosis				0.011
2006–2008	290 (16.6)	20 (11.0)	151 (18.8)	
2009–2012	651 (37.2)	68 (37.4)	253 (31.4)	
2013–2016	808 (46.2)	94 (51.6)	401 (49.8)	
Age, years, mean (SD)	66.1 (10.7)	60.4 (10.9)	61.9 (10.9)	<0.001
Age, years, range				<0.001
18–55	236 (13.5)	56 (30.8)	192 (23.9)	
56–69	797 (45.6)	93 (51.1)	403 (50.1)	
70+	716 (40.9)	33 (18.1)	210 (26.1)	
Gender				0.001
Male	817 (46.7)	63 (34.6)	334 (41.5)	
Female	932 (53.3)	119 (65.4)	471 (58.5)	
Race				0.165
White	1373 (78.5)	155 (85.2)	617 (76.6)	
Black	116 (6.6)	8 (4.4)	48 (6.0)	
Hispanic	88 (5.0)	9 (4.9)	46 (5.7)	
Asian Pacific Islander	88 (5.0)	4 (2.2)	56 (7.0)	
Other/Missing	84 (4.8)	6 (3.3)	38 (4.7)	
Insurance Status				<0.001
Uninsured	35 (2.0)	1 (0.5)	9 (1.1)	
Private	616 (35.2)	78 (42.9)	386 (48.0)	
Public/Government	1048 (59.9)	97 (53.3)	395 (49.1)	
Missing	50 (2.9)	6 (3.3)	15 (1.9)	
Charlson comorbidity score				<0.001
0–1	1525 (87.2)	171 (94.0)	735 (91.3)	
≥2	224 (12.8)	11 (6.0)	70 (8.7)	
Facility Type				<0.001
Community Cancer Program, Comprehensive Community Cancer Program, or Integrated Network Cancer Program	585 (33.4)	45 (24.7)	311 (38.6)	
Academic/Research Program	1137 (65.1)	129 (70.9)	465 (57.8)	
Other/Unknown	27 (1.5)	8 (4.4)	29 (3.6)	
Tumor size, cm				<0.001
<2	127 (7.3)	4 (2.2)	39 (4.8)	
2–5	766 43.8)	38 (20.9)	297 (36.9)	
>5	767 (43.9)	116 (63.7)	400 (49.7)	
Missing	89 (5.1)	24 (13.2)	69 (8.6)	
Tumor grade				<0.001
Well-differentiated	216 (12.3)	16 (8.8)	79 (9.8)	
Moderately differentiated	896 (51.2)	78 (42.9)	375 (46.6)	
Poorly or undifferentiated	415 (23.7)	48 (26.4)	241 (29.9)	
Missing	222 (12.7)	40 (22.0)	110 (13.7)	
TNM Clinical T				<0.001
T1	781 (44.7)	57 (31.3)	251 (31.2)	
T2	276 (15.8)	57 (31.3)	178 (22.1)	
T3	84 (4.8)	15 (8.2)	84 (10.4)	
T4	13 (0.7)	3 (1.6)	5 (0.6)	
Tx	595 (34.0)	50 (27.5)	287 (35.7)	
TNM Clinical N				0.186
N0	1353 (77.4)	139 (76.4)	606 (75.3)	
N1	29 (1.7)	6 (3.3)	24 (3.0)	
Nx	367 (21.0)	37 (20.3)	175 (21.7)	
Surgery type				<0.001
Wedge, segmentectomy, or sectionectomy	899 (51.4)	82 (45.1)	372 (46.2)	
Hemi-hepatectomy	607 (34.7)	56 (30.8)	272 (33.8)	
Extended hepatectomy	217 (12.4)	36 (19.8)	130 (16.1)	
Surgery NOS	26 (1.5)	8 (4.4)	31 (3.9)	
Resection Margin				<0.001
Negative	1446 (82.7)	132 (72.5)	504 (62.6)	
Positive	212 (12.1)	37 (20.3)	242 (30.1)	
Missing	91 (5.2)	13 (7.1)	59 (7.3)	
Regional nodes positive				<0.001
0	743 (42.5)	80 (44.0)	386 (48.0)	
≥1	75 (4.3)	9 (4.9)	63 (7.8)	
No LN examined	919 (52.5)	89 (48.9)	342 (42.5)	
Unknown	12 (0.7)	4 (2.2)	14 (1.7)	
Number of lymph nodes examined				<0.001
0	919 (52.5)	89 (48.9)	342 (42.5)	
1–5	627 (35.8)	72 (39.6)	334 (41.5)	
≥6	176 (10.1)	17 (9.3)	102 (12.7)	
Unknown	27 (1.5)	4 (2.2)	27 (3.4)	
Chemotherapy regimen				<0.001
None	1749 (100)	0 (0)	0 (0)	
Single-agent	0 (0)	34 (18.7)	404 (50.2)	
Multi-agent	0 (0)	134 (73.6)	345 (42.9)	
Missing	0 (0)	14 (7.7)	56 (7.0)	
Radiation				<0.001
No	1689 (96.6)	144 (79.1)	495 (61.5)	
Yes	60 (3.4)	38 (20.9)	310 (38.5)	

**Table 2:** Univariable and multivariable logistic regression analyses for factors associated with use of neoadjuvant therapy.

	Univariable		Multivariable	
Characteristic	OR (95% CI)	*p* Value	OR (95% CI)	*p* Value
Year of diagnosis				
2006–2008				
2009–2012	1.66 (0.99–2.77)	0.052	**2.09 (1.19–3.68)**	**0.011**
2013–2016	1.71 (1.05–2.81)	0.033	**2.41 (1.37–4.24)**	**0.002**
Age, years, range				
18–55				
56–69	0.59 (0.42–0.84)	0.003	**0.55 (0.36–0.83)**	**0.004**
70+	0.27 (0.17–0.43)	<0.001	**0.20 (0.12–0.34)**	**<0.001**
Gender				
Male				
Female	1.55 (1.13–2.12)	0.007	**1.46 (1.05–2.04)**	**0.025**
Race				
White				
Black	0.63 (0.30–1.30)	0.208	0.52 (0.24–1.13)	0.097
Hispanic	0.86 (0.43–1.73)	0.676	0.71 (0.34–1.51)	0.379
Asian Pacific Islander	0.36 (0.13–0.98)	0.045	0.32 (0.12–0.92)	0.033
Other/Missing	0.63 (0.27–1.46)	0.281	0.51 (0.21–1.26)	0.143
Insurance Status				
Uninsured	0.29 (0.04–2.15)	0.227	0.24 (0.03–1.90)	0.177
Private				
Public/Government	0.86 (0.63–1.18)	0.352	1.63 (1.12–2.38)	0.012
Charlson comorbidity score				
0–1				
≥2	0.49 (0.27–0.92)	0.026	0.56 (0.29–1.06)	0.076
Facility Type				
Community Cancer Program, Comprehensive Community Cancer Program, or Integrated Network Cancer Program				
Academic/Research Program	1.6 (1.13–2.27)	0.008	**1.84 (1.26–2.69)**	**0.002**
Tumor size, cm				
<2				
2–5	1.48 (0.52–4.21)	0.459	1.35 (0.46–3.92)	0.584
>5	4.13 (1.5–11.32)	0.006	**3.8 (1.34–10.72)**	**0.012**
Clinical stage				
I				
II	2.08 (1.41–3.06)	<0.001	**1.84 (1.22–2.78)**	**0.004**
IIIA	1.48 (0.80–2.71)	0.209	1.38 (0.72–2.63)	0.329
IIIB	2.74 (1.24–6.04)	0.013	**3.28 (1.30–8.24)**	**0.012**
Radiation				
No				
Yes	1.56 (1.07–2.26)	0.020	1.28 (0.85–1.93)	0.245

**Table 3:** Univariable and multivariable logistic regression analyses for factors associated with use of adjuvant therapy.

	Univariable		Multivariable	
Characteristic	OR (95% CI)	*p* Value	OR (95% CI)	*p* Value
Year of diagnosis				
2006–2008				
2009–2012	0.75 (0.58–0.95)	0.019	1.15 (0.84–1.57)	0.395
2013–2016	0.95 (0.76–1.20)	0.683	1.63 (1.20–2.25)	0.002
Age, years, range				
18–55				
56–69	0.62 (0.50–0.78)	<0.001	**0.69 (0.51–0.92)**	**0.012**
70+	0.36 (0.28–0.46)	<0.001	**0.39 (0.28–0.55)**	**<0.001**
Gender				
Male				
Female	1.24 (1.04–1.46)	0.014	**1.43 (1.16–1.75)**	**0.001**
Race				
White				
Black	0.92 (0.65–1.31)	0.644	0.74 (0.47–1.16)	0.193
Hispanic	1.16 (0.80–1.68)	0.422	0.98 (0.61–1.58)	0.945
Asian Pacific Islander	1.42 (1.00–2.01)	0.050	1.47 (0.96–2.23)	0.074
Other/Missing	1.01 (0.68–1.49)	0.974	1.00 (0.62–1.60)	0.996
Insurance Status				
Uninsured	0.41 (0.20–0.86)	0.019	0.44 (0.19–1.03)	0.059
Private				
Public/Government	0.60 (0.51–0.71)	<0.001	0.85 (0.67–1.08)	0.189
Charlson comorbidity score				
0–1				
≥2	0.65 (0.49–0.86)	0.003	0.76 (0.54–1.06)	0.107
Facility Type				
Community Cancer Program, Comprehensive Community Cancer Program, or Integrated Network Cancer Program				
Academic/Research Program	0.77 (0.65–0.92)	0.003	**0.69 (0.55–0.86)**	**0.001**
Tumor size, cm				
<2				
2–5	1.26 (0.86–1.85)	0.233	1.53 (0.96–2.44)	0.077
>5	1.70 (1.16–2.48)	0.006	**1.93 (1.21–3.07)**	**0.006**
Tumor grade				
Well-differentiated				
Moderately differentiated	1.14 (0.86–1.52)	0.353	0.81 (0.57–1.15)	0.238
Poorly or undifferentiated	1.59 (1.17–2.15)	0.003	1.11 (0.76–1.61)	0.598
Pathologic stage				
I				
II	2.3 (1.82–2.91)	<0.001	**2.50 (1.87–3.34)**	**<0.001**
IIIA	3.21 (2.39–4.33)	<0.001	**3.36 (2.32–4.84)**	**<0.001**
IIIB	3.51 (2.33–5.28)	<0.001	**3.33 (1.94–5.72)**	**<0.001**
Surgery type				
Wedge, segmentectomy, or sectionectomy				
Hemi-hepatectomy	1.08 (0.90–1.31)	0.404	1.08 (0.86–1.35)	0.531
Extended hepatectomy	1.45 (1.13–1.86)	0.004	1.06 (0.78–1.45)	0.710
Surgery NOS	2.88 (1.69–4.92)	<0.001	1.90 (0.98–3.69)	0.058
Resection Margin				
Negative				
Positive	3.27 (2.65–4.04)	<0.001	**1.83 (1.41–2.39)**	**<0.001**
Regional lymph nodes examined				
No				
Yes	1.46 (1.23–1.73)	<0.001	1.12 (0.88–1.42)	0.351
Radiation				
No				
Yes	17.63 (13.14–23.66)	<0.001	**16.81 (12.15–23.26)**	**<0.001**

## Data Availability

The data can be shared up on request.

## Supplementary Materials

The following supporting information can be downloaded at: https://www.mdpi.com/article/10.3390/cancers14174320/s1, Figure S1: Comparison of overall survival by treatment strategy using multivariable Cox proportional-hazards regression analyses for patients with resectable intrahepatic cholangiocarcinoma; Figure S2: Comparison of overall survival by treatment strategy adjusted using Cox regression with IPTW and three-month landmark analyses for patients with (**a**) clinical stage I and (**b**) stages II-III resectable intrahepatic cholangiocarcinoma; Table S1: Baseline Demographic and Tumor Characteristics for complete ICC patient cohort. NAT: neoadjuvant therapy; AT: adjuvant therapy.
