# Supplementary material for: Systemic Therapy Is Associated with Improved Oncologic Outcomes in Resectable Stage II/III Intrahepatic Cholangiocarcinoma: An Examination of the National Cancer Database over the Past Decade
Source: Cancers (Basel). 2022 Sep 3;14(17):4320. doi: 10.3390/cancers14174320 (PMC9454548; doi:10.3390/cancers14174320)

Supplementary Table S1. Baseline Demographic and Tumor Characteristics for complete ICC patient cohort. NAT: neoadjuvant therapy; AT: adjuvant therapy.

Characteristic	N (%)
Age, years, mean (SD)	64.5 (10.9)
Age, years, range	
18-55	484 (17.7)
56-69	1293 (47.3)
70+	959 (35.1)
Median follow up, months (IQR)	30.8 (16.6–53.0)
Gender	
Male	1214 (44.4)
Female	1522 (55.6)
Race	
White	2145 (78.4)
Black	172 (6.3)
Hispanic	143 (5.2)
Asian Pacific Islander	148 (5.4)
Other/Missing	128 (4.7)
Insurance Status, n (%)	
Uninsured	45 (1.6)
Private	1080 (39.5)
Public/Government	1540 (56.3)
Missing	71 (2.6)
Charlson comorbidity score	
0-1	2431 (88.9)
≥2	305 (11.1)
Facility Type	
Community Cancer Program, Comprehensive Community Cancer Program, or Integrated Network Cancer Program	941 (34.4)
Academic / Research Program	1731 (63.3)
Other/Unknown	64 (2.3)
Tumor size, cm	
<2	170 (6.2)
2-5	1101 (40.2)
>5	1283 (46.9)
Missing	182 (6.7)
Tumor grade	
Well-differentiated	311 (11.4)
Moderately differentiated	1349 (49.3)
Poorly or undifferentiated	704 (25.7)
Missing	372 (13.6)
TNM Clinical T	
T1	1089 (39.8)

T2	511 (18.7)
T3	183 (6.7)
T4	21 (0.8)
Tx	932 (34.1)
TNM Clinical N	
N0	2098 (76.7)
N1	59 (2.2)
Nx	579 (21.2)
Surgery type	
Wedge, segmentectomy, or sectionectomy	1353 (49.5)
Hemi-hepatectomy	935 (34.2)
Extended hepatectomy	383 (14)
Surgery NOS	65 (2.4)
Resection Margin	
Negative	2082 (76.1)
Positive	491 (17.9)
Missing	163 (6.0)
Regional nodes positive	
0	1209 (44.2)
≥1	147 (5.4)
No LN examined	1350 (49.3)
Unknown	30 (1.1)
Number of lymph nodes examined	
0	1350 (49.3)
1-5	1033 (37.8)
≥6	295 (10.8)
Unknown	58 (2.1)
Treatment strategy	
NAT*	182 (6.7)
Surgery alone	1749 (63.9)
AT†	805 (29.4)
Chemotherapy regimen	
None	1749 (63.9)
Single-agent	438 (16)
Multi-agent	479 (17.5)
Missing	70 (2.6)
Radiation	
No	2328 (85.1)
Yes	408 (14.9)

Supplementary Figure S1. Comparison of overall survival by treatment strategy using multivariable Cox proportional-hazards regression analyses for patients with resectable intrahepatic cholangiocarcinoma.

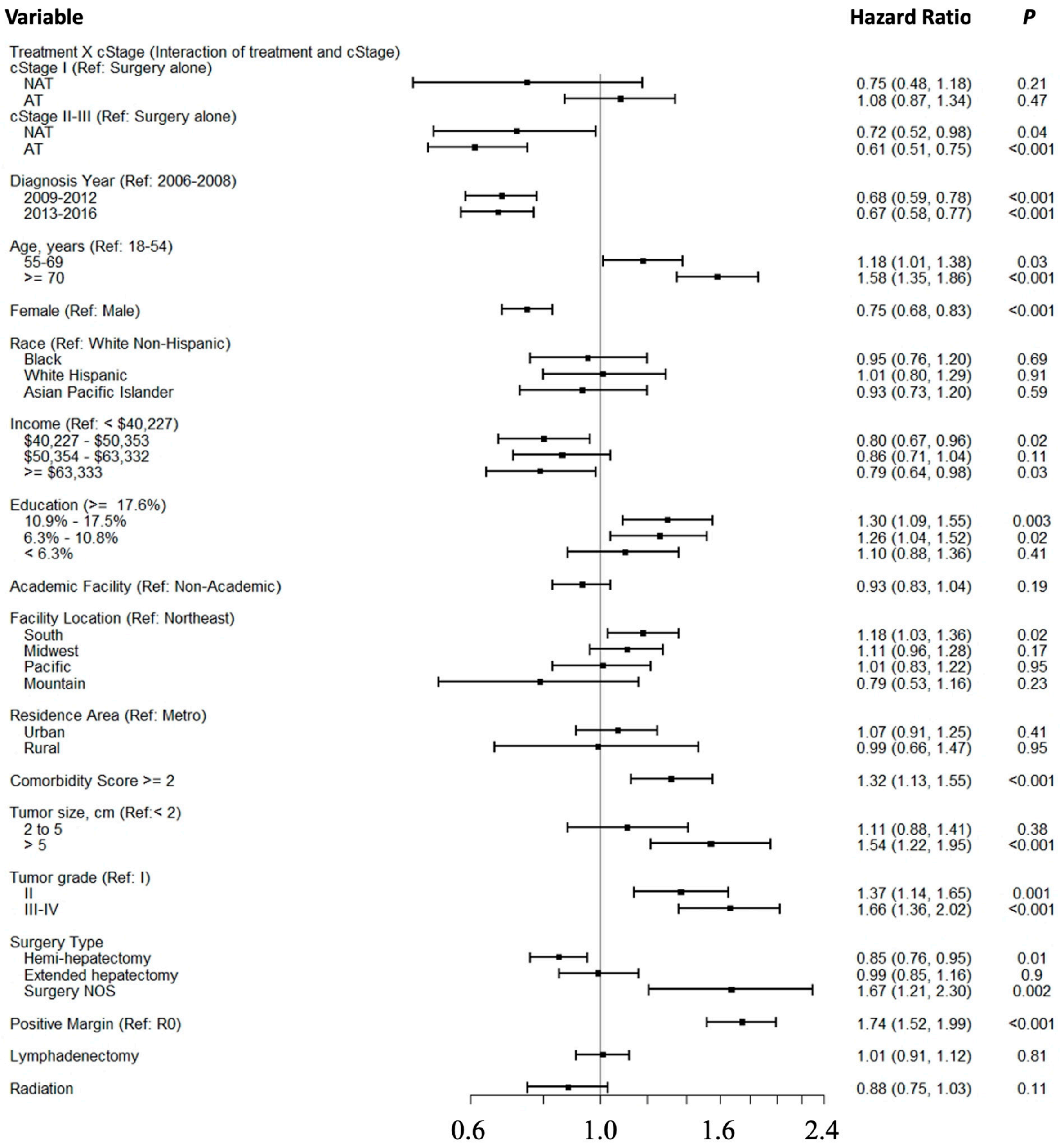

Supplementary Figure S2. Comparison of overall survival by treatment strategy adjusted using Cox regression with IPTW and three-month landmark analyses for patients with (a) clinical stage I and (b) stages II-III resectable intrahepatic cholangiocarcinoma. IPTW: inverse probability of treatment weighting.

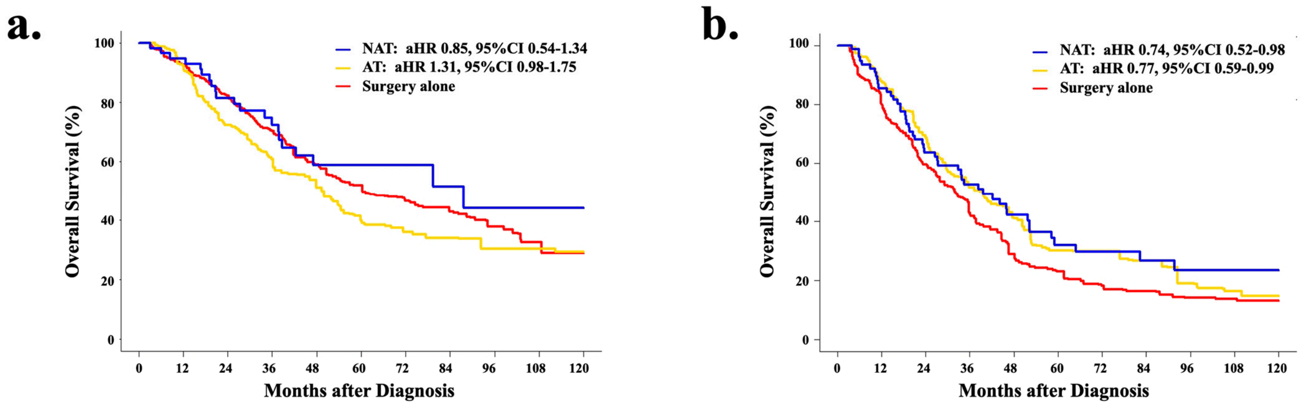

Supplement: Supplementary file 1 [file cancers-14-04320-s001.zip › cancers-1847323-supplementary.pdf]
